# *Enterobacteriaceae* are essential for the modulation of colitis severity by fungi

**DOI:** 10.1186/s40168-018-0538-9

**Published:** 2018-09-01

**Authors:** Bruno Sovran, Julien Planchais, Sarah Jegou, Marjolene Straube, Bruno Lamas, Jane Mea Natividad, Allison Agus, Louise Dupraz, Jérémy Glodt, Grégory Da Costa, Marie-Laure Michel, Philippe Langella, Mathias L. Richard, Harry Sokol

**Affiliations:** 1 Micalis Institute, INRA, AgroParisTech, Université Paris-Saclay, Domaine de Vilvert, Jouy-en-Josas, France; 2 Sorbonne Universités, École Normale Supérieure, CNRS, INSERM, APHP Laboratoire des Biomolécules (LBM), 27 rue de Chaligny, Paris, France; 3 Department of Gastroenterology, Saint Antoine Hospital, 184 rue du Faubourg Saint-Antoine, Paris, France

**Keywords:** *Enterobacteriaceae*, *S. boulardii* CNCM I-745, *C. albicans*, Colitis, Microbiota, Fungi

## Abstract

**Background:**

Host-microbe balance maintains intestinal homeostasis and strongly influences inflammatory conditions such as inflammatory bowel diseases (IBD). Here we focused on bacteria-fungi interactions and their implications on intestinal inflammation, a poorly understood area.

**Methods:**

Dextran sodium sulfate (DSS)-induced colitis was assessed in mice treated with vancomycin (targeting gram-positive bacteria) or colistin (targeting *Enterobacteriaceae*) and supplemented with either *Saccharomyces boulardii* CNCM I-745 or *Candida albicans*. Inflammation severity as well as bacterial and fungal microbiota compositions was monitored.

**Results:**

While *S. boulardii* improved DSS-induced colitis and *C. albicans* worsened it in untreated settings, antibiotic treatment strongly modified DSS susceptibility and effects of fungi on colitis. Vancomycin-treated mice were fully protected from colitis, while colistin-treated mice retained colitis phenotype but were not affected anymore by administration of fungi. Antibacterial treatments not only influenced bacterial populations but also had indirect effects on fungal microbiota. Correlations between bacterial and fungal relative abundance were dramatically decreased in colistin-treated mice compared to vancomycin-treated and control mice, suggesting that colistin-sensitive bacteria are involved in interactions with fungi. Restoration of the *Enterobacteriaceae* population by administrating colistin-resistant *Escherichia coli* reestablished both beneficial effects of *S. boulardii* and pathogenic effects of *C. albicans* on colitis severity. This effect was at least partly mediated by an improved gut colonization by fungi.

**Conclusions:**

Fungal colonization of the gut is affected by the *Enterobacteriaceae* population, indirectly modifying effects of mycobiome on the host. This finding provides new insights into the role of inter-kingdom functional interactions in intestinal physiopathology and potentially in IBD.

**Electronic supplementary material:**

The online version of this article (10.1186/s40168-018-0538-9) contains supplementary material, which is available to authorized users.

## Background

The gastrointestinal tract (GIT) is composed of a complex association of epithelial cells, immune cells, food antigens, and microorganisms. The mammalian GIT is colonized by diverse commensal microbial communities consisting of bacteria, fungi, and viruses [1]. Alteration of the composition of function of the gut microbiota, i.e., dysbiosis, has been associated with several human diseases and particularly with inflammatory bowel disease (IBD). The bacterial microbiota in IBD has been thoroughly investigated; several groups worldwide have observed a reduction in biodiversity, a decreased relative abundance of bacteria belonging to the phylum Firmicutes (such as *Faecalibacterium prausnitzii*), and an increased relative abundance of bacteria belonging to the phylum Proteobacteria (such as *Escherichia coli*) [2–6]. Recently, we showed that the fungal microbiota is similarly skewed in IBD, characterized by an increased Basidiomycota/Ascomycota ratio, a decreased proportion of *Saccharomyces cerevisiae*, and an increased proportion of *Candida albicans* in IBD patients compared with healthy subjects [7]. Interestingly, these two yeasts were reported to have opposite impacts on intestinal inflammation. The probiotic strain *S. boulardii* CNCM I-745, which belongs to the species *S. cerevisiae* [8], protects against pathogen-associated diarrhea and colitis in both murine models and humans [9–11] and has a beneficial effect on IBD in murine models [12, 13]. On the other hand, *C. albicans*, which is the most prevalent fungus in the human intestinal microbiota [14, 15], shows improved colonization in inflammatory context and simultaneously worsens intestinal inflammation in a murine colitis model [16].

Antibiotics used in the treatment of bacterial infections indiscriminately kill pathogens and commensal microbes in the gut, leading to dysbiosis [17]. Moreover, it has been shown for decades that broad-spectrum antibiotics and antibiotics targeting anaerobic bacteria induce differential effects on susceptibility to fungal infection and, notably, predispose patients to vaginal mucosal infections by *C. albicans* [18]*.* Broad-spectrum antibiotic treatment triggers a strong increase in gut fungi loads in a mouse model [19], which has commonly been interpreted as resulting mainly from the liberation of ecological niches for fungi proliferation. However, fungal-bacterial interactions and their role in a physiopathological context have been poorly studied to date. We have previously showed that both the bacterial and fungal microbiota are dysbiotic in IBD and that bacterial-fungal inter-kingdom interactions are altered in ulcerative colitis and Crohn’s disease compared to healthy conditions [7].

Here, we used *C. albicans* and *S. boulardii* CNCM I-745, as model fungi, to explore the potential role of bacterial components in mediating the effects of fungi in intestinal inflammation. We showed that the presence of bacterial species belonging to the *Enterobacteriaceae* family greatly influences the fitness of *C. albicans* and *S. boulardii* in the gastrointestinal tract and further determines how these fungi modulate the outcome of intestinal inflammation. Overall, the results demonstrate the crucial role of inter-kingdom functional interactions in intestinal physiopathology.

## Methods

### Mice

For the conventional experiment, female C57BL/6J mice were purchased from Janvier (France) and used 1 week after delivery. All experiments were done with appropriate control groups (H_2_O), from the same batch of mice. Mice were kept 1 week in the same room prior starting treatments. Ten-week-old mice were fed ad libitum with Ssniff control diet V112x M-Z (Ssniff Spezialdiaten, Germany). All experiments were performed in accordance with the Comite d’Ethique en Experimentation Animale (COMETHEA C2EA – 45, Jouy en Josas, France). Every experiment was repeated two or three times.

### Antibiotic treatment and induction of colitis with dextran sodium sulfate (DSS)

A broad-spectrum antibiotic cocktail was prepared by mixing ampicillin (2 g/L), neomycin (2 g/L), metronidazole (2 g/L), and vancomycin (1 g/L) in drinking water. To selectively deplete specific bacteria, we treated the mice until sacrifice with vancomycin (1 g/L in drinking water) or colistin (600 μg/gavage; Sigma). One week after starting the antibiotic treatment, mice were given 2% (wt/vol) DSS (molecular weight, 36,000–50,000; MP Biomedicals, Solon, OH) dissolved in sterile drinking water ad libitum for 7 days, followed by a recovery period (water only) of 5 days. Animals were monitored daily for weight loss and disease activity index (including three parameters: weight loss, stool consistency, and presence of blood in feces).

### Gavage with fungi and *E. coli* strains

*S. boulardii* CNCM I-745 (syn. HANSEN CBS 5926, Biocodex Laboratories, Gentilly, France) and *C. albicans* SC5314 (ATCC, Molsheim, France) were used in this study. Both yeasts were grown on yeast extract peptone dextrose (YEPD) medium overnight at 37 °C. Aliquots containing a yeast suspension of 5 × 10^7^ CFU/mL were prepared and stored at − 80 °C. A yeast suspension (10^7^ CFU in 200 μL) or control medium was administered daily to 10-week-old mice by intragastric gavage.

The *E. coli* MCR1 strain, which is rendered resistant to colistin by the mobilized colistin resistance (MCR) gene, was used in this study. This strain is a veterinary isolate commensal [20] and was provided by Thomas Guillard (CHU Reims, Hôpital Robert Debré, Laboratoire de Bactériologie-Virologie-Hygiène, F-51092 Reims, France). The bacteria were grown in LB at 37 °C overnight and then aliquoted at 5 × 10^8^ CFU/mL. A bacterial suspension (10^8^ CFU in 200 μL) or control medium was administered daily to 10-week-old mice by intragastric gavage.

### Histology

Colon samples for histological studies were fixed in 4% paraformaldehyde (Electron Microscopy Sciences, Hatfield, PA, USA), embedded in paraffin, and then stained with hematoxylin and eosin (Sigma-Aldrich, Saint Louis, USA) for histological scoring. Histological scoring was performed blinded according to the method previously described [21].

### Gene expression analysis using quantitative reverse-transcription PCR (qRT-PCR)

Total RNA was isolated from colon samples using an RNeasy Mini Kit (Qiagen, Hilden, Germany), including a DNAse treatment step, according to the manufacturer’s instructions. Quantitative RT-PCR was performed using SuperScript II Reverse Transcriptase (Life Technologies, Saint Aubin, France) followed by a Takyon SYBR Green PCR kit (Eurogentec, Liège, Belgium) in a StepOnePlus apparatus (Applied Biosystems, Foster City, CA, USA) with specific mouse oligonucleotides. The oligonucleotides used were as follows: GAPDH—sense: 5′-AACTTTGGCATTGTGGAAGG-3′; antisense: 5′-ACACATTGGGGGTAGGAACA-3′, Reg3g—sense: 5′-TTCCTGTCCTCCATGATCAAAA-3′; antisense: 5′-CATCCACCTCTGTTGGGTTCA-3′. We used the 2^−ΔΔCt^ quantification method with mouse GAPDH as an endogenous control and calibrated the assay to the wild type.

### Cytokine quantification

The colonic explants were cultured (37 °C, 10% CO_2_) overnight in 24-well tissue culture plates (Costar, Corning, Amsterdam, the Netherlands) in l mL of complete RPMI 1640 medium. The culture supernatants were collected and stored at − 80 °C until processing. ELISA was performed on the supernatants according to the manufacturer’s instructions in order to quantify IFN-γ (Mabtech, Nacka Strand, Sweden). For the colonic explants, cytokine concentrations were normalized according to the weight of each colonic explant.

### Quantification of fecal lipocalin (LCN2) levels

Frozen fecal samples were weighed and suspended in cold PBS. Samples were then agitated on a FastPrep (MP Biomedicals, Santa Ana, USA) bead beating machine for 40 s on setting 6 using 4.5-mm glass beads to obtain a homogenous fecal suspension. Samples were then centrifuged for 5 min at 10,000*g* (4 °C), and clear supernatants were collected and stored at − 20 °C until analysis. LCN2 levels were estimated using a DuoSet murine LCN2 ELISA kit (R&D Systems, Minneapolis, USA) as per the manufacturer’s instructions and expressed as pg/mg of stool.

### Quantification of fungi and bacteria in fresh stools

Fresh stools were collected over the course of the study to determine the quantities of yeast and bacteria remaining after intragastric gavage. Fresh stools were weighed and suspended in cold PBS (3 μL/mg of feces). Tenfold serial dilutions were performed until the desired concentrations were reached. For fungi quantification, diluted feces were plated on YEPD agar plates supplemented with ampicillin (100 mg/mL) and incubated at 30 °C. After 2 days of growth, fungi were counted, and the absolute quantities of yeast were determined according to the corresponding dilutions. For *E. coli* MCR1 (resistant to colistin) quantification, diluted feces were plated on LB agar plates supplemented with colistin (4 mg/L) and incubated at 37 °C. After 1 day of growth, bacteria were counted, and the absolute quantities of *E. coli* were determined according to the corresponding dilutions.

### Fecal DNA extraction and fungal quantification via quantitative PCR (qPCR)

Fecal DNA was extracted from weighed stool samples as previously described [22]. More precisely, the fecal samples were weighed and then resuspended for 10 min at room temperature in 250 μL of 4 M guanidine thiocyanate in 0.1 M Tris (pH 7.5) (Sigma-Aldrich, Saint Louis, USA) and 40 μL of 10% *N*-lauroyl sarcosine (Sigma-Aldrich, Saint Louis, USA). After the addition of 500 μL of 5% *N*-lauroyl sarcosine in 0.1 M phosphate buffer (pH 8.0), the 2-mL tubes were incubated at 70 °C for 1 h. One volume (750 mL) of a mixture of 0.1- and 0.6-mm-diameter silica beads (Sigma-Aldrich, Saint Louis, USA) (previously sterilized by autoclaving) was added, and the tube was shaken at 6.5 m/s in three bouts of 30 s each in a FastPrep (MP Biomedicals, Santa Ana, USA) apparatus. Polyvinylpolypyrrolidone (15 mg) (Sigma-Aldrich, Saint Louis, USA) was added to the tube, which was then vortexed and centrifuged for 5 min at 20,000*g*. After recovery of the supernatant, the pellets were washed with 500 μL of TENP (50 mM Tris (pH 8), 20 mM EDTA (pH 8), 100 mM NaCl, 1% polyvinylpolypyrrolidone) and centrifuged for 5 min at 20,000*g*, and the new supernatant was added to the first supernatant. The washing step was repeated two times. The pooled supernatant (approximately 2 mL) was briefly centrifuged to remove particulate matter and then split into two 2-mL tubes. Nucleic acids were precipitated by the addition of 1 volume of isopropanol for 10 min at room temperature and centrifugation for 10 min at 20,000*g*. The pellets were resuspended and pooled in 450 μL of 100 mM phosphate buffer, pH 8, and 50 mL of 5 M potassium acetate. The tube was placed on ice overnight and centrifuged at 20,000*g* for 30 min. The supernatant was then transferred to a new tube containing 20 μL of RNase (1 mg/mL) and incubated at 37 °C for 30 min. Nucleic acids were precipitated by the addition of 50 μL of 3 M sodium acetate and 1 mL of absolute ethanol. The tube was incubated for 10 min at room temperature, and the nucleic acids were recovered by centrifugation at 20,000*g* for 15 min. The DNA pellet was finally washed with 70% ethanol, dried, and resuspended in 100 μL of Tris–EDTA (TE) buffer. The DNA suspensions were stored at − 20 °C for real-time qPCR analysis of the 16S rDNA or ITS2 sequences. DNA was then subjected to qPCR by using a Takyon SYBR Green PCR kit (Eurogentec, Maastricht, The Netherlands) for quantification of all fungal sequences or by using TaqMan Gene Expression Assays (Life Technologies) for quantification of all bacterial sequences. The probes and primers for the bacterial 16S DNA genes and primers for the fungal 18S DNA genes were as described previously [23]. Reference standard (with quantified *C. albicans* DNA) was used in the same qPCR, allowing the quantification of the CFU in each sample. The threshold cycle for each sample was determined for each gene and was normalized to the CT value of the all-bacteria 16S ribosomal RNA gene. Data were calculated using the 2^−ΔΔCt^ method.

### 16S DNA gene and ITS2 sequencing

DNA was isolated from the feces of mice before and after DSS treatment using the protocol described above. Bacterial diversity was determined for each sample by targeting a portion of the ribosomal genes. A 16S DNA gene fragment comprising the V3 and V4 hypervariable regions (16S (sense) 5′-TACGGRAGGCAGCAG-3′ and (antisense) 5′-CTACCNGGGTATCTAAT-3′) was amplified using an optimized and standardized 16S-amplicon-library preparation protocol (Metabiote, GenoScreen, Lille, France). Briefly, 16S DNA gene PCR was performed using 5 ng of genomic DNA according to the manufacturer’s protocol (Metabiote) using 192 bar-coded primers (Metabiote MiSeq Primers) at final concentrations of 0.2 μM and an annealing temperature of 50 °C for 30 cycles. The PCR products were purified using an Agencourt AMPure XP-PCR Purification system (Beckman Coulter, Brea, CA, USA), quantified according to the manufacturer’s protocol, and multiplexed at equal concentrations. Sequencing was performed using a 300-bp paired-end sequencing protocol on an Illumina MiSeq platform (Illumina, San Diego, CA, USA) at GenoScreen, Lille, France. Raw paired-end reads were subjected to the following processes: (1) quality-filtering using the PRINSEQ-lite PERL script [24] by truncating from the 3′ end those bases that did not exhibit a quality < 30 based on the Phred algorithm; (2) paired-end read assembly using FLASH [25] (fast length adjustment of short reads to improve genome assemblies) with a minimum overlap of 30 bases and a 97% overlap identity; and (3) searching for and removing both forward and reverse primer sequences using CutAdapt, with no mismatches allowed in the primer sequences. Assembled sequences for which perfect forward and reverse primers were not found were eliminated. A similar approach was used for fungi microbiota using the primers ITS2 (sense) 5′-GTGARTCATCGAATCTTT-3′ and (antisense) 5′-GATATGCTTAAGTTCAGCGGGT-3′ and the optimized and standardized ITS2-amplicon-library preparation protocol (Metabiote, GenoScreen).

### 16S and ITS2 sequence analysis

The sequences were demultiplexed and quality filtered using the QIIME version 1.8.0 software package [26]. The sequences were then assigned to OTUs using the UCLUST algorithm [27] with a 97% pairwise identity threshold and classified taxonomically using the Greengenes reference database (version 13.5) for bacteria and the UNITE ITS database (alpha version 12_11) for fungi [28]. Rarefaction was performed (20,000 and 250 sequences per sample for 16S and ITS2, respectively) and used to compare the relative abundance of OTUs across samples. Alpha diversity was estimated using the Shannon diversity index or the number of observed species. Beta diversity was measured by a Bray–Curtis distance matrix and was used to build principal coordinates analysis (PCoA) plots. The linear discriminant analysis (LDA) effect size (LEfSe) algorithm was used to identify taxa that were specific to diet and/or treatment. Deposition of the raw sequence data in the European Nucleotide Archive is in process; the accession number is pending.

### Statistics

GraphPad Prism version 6.0 (San Diego, CA, USA) was used for all analyses and preparation of graphs. For all data displayed in graphs, the results are expressed as the mean ± s.e.m. (*n* = 5 to 12 per group). For comparisons between two groups, a two-tailed Student’s *t* test for unpaired data or a nonparametric Mann–Whitney test was used. For comparisons among more than two groups, one-way analysis of variance (ANOVA) and a post hoc Tukey test or a nonparametric Kruskal–Wallis test followed by a post hoc Dunn’s test was used. For all statistical tests, differences with a *P* value less than 0.05 were considered significant.

Correlation between bacterial and fungal taxon relative abundance was measured by distance correlation [29] and as described previously [7]. Only taxa present in at least 50% of the samples of each group were included. In addition to the distance correlation, the sign of Spearman’s correlation was computed to describe heuristically the direction of association between microbial taxa. The distance correlation was computed in R-3.2.3 using the package energy v1.6.2. The *P* values were corrected using the Benjamini–Hochberg procedure to control the false discovery rate (*P* < 0.25).

## Results

### *C. albicans* and *S. boulardii* have opposite effects on DSS-induced colitis in untreated mice but lose their effects after broad-spectrum antibiotic treatment

**Fig. 1:**
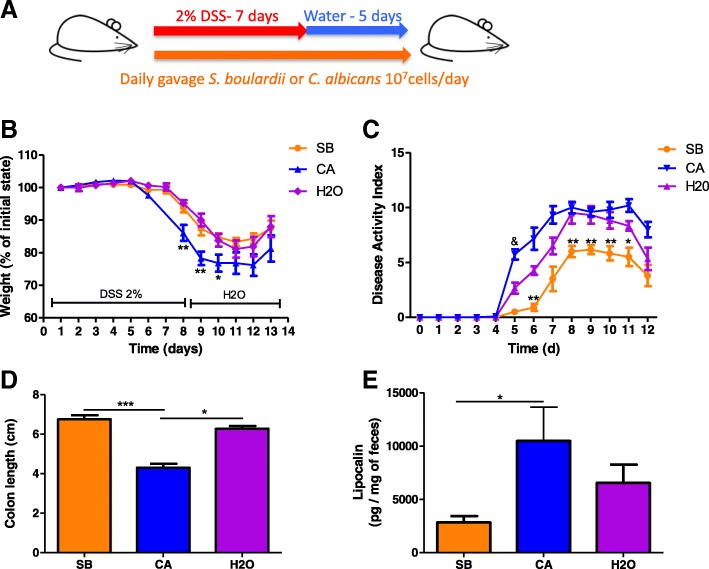
In untreated mice, *S. boulardii* and *C. albicans* have opposite effects on DSS-induced colitis. **a** Experimental design for the administration of fungi (*C. albicans* and *S. boulardii*) and dextran sulfate sodium (DSS) administration in conventional mice. **b** Weight of DSS-exposed mice. For statistical comparisons, an asterisk (*) indicates *C. albicans* (CA) versus H_2_O. **c** Disease activity index (DAI) of DSS-exposed mice. For statistical comparisons, an asterisk (*) indicates *S. boulardii* versus H_2_O, and (&) indicates *C. albicans* versus H_2_O. **d** Length of the colons of mice treated with *C. albicans* (CA), *S. boulardii* (SB), and vehicle (H_2_O). **e** Secreted lipocalin (pg/mg of feces) in the feces of mice treated with *C. albicans* (CA), *S. boulardii* (SB), and untreated controls (H_2_O). Throughout, data are given as the mean ± s.e.m. *P < 0.05, **P < 0.01, and ^&&&^*P* < 0.001 by one-way ANOVA with a post hoc Tukey or Dunn’s test; *n* = 12 mice per group from two independent experiments

We first evaluated the effect of daily administration of *C. albicans* and *Saccharomyces boulardii* CNCM I-745 on dextran sodium sulfate (DSS)-induced colitis (Fig. 1a). As previously described [16], administration of *C. albicans* induced an increased severity of colitis at D7, characterized by significant weight loss (Fig. 1b), higher disease activity index (DAI) (Fig. 1c), colon shortening (Fig. 1d), and increased lipocalin levels (Fig. 1e). On the other hand, *S. boulardii* showed an anti-inflammatory effect, reducing most symptoms of colitis evaluated by the DAI (Fig. 1b–d), with reduced levels of lipocalin (Fig. 1e).

**Fig. 2:**
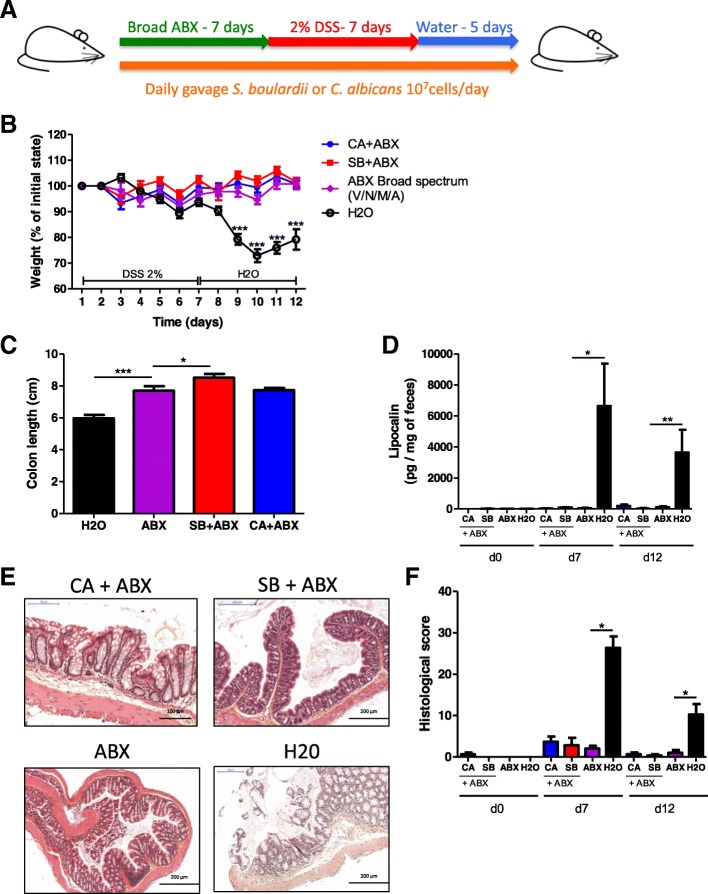
Broad-spectrum ABX protect against DSS-induced colitis, and fungi have limited effects on DSS-induced colitis in their presence. **a** Experimental design for the administration of broad-spectrum antibiotics (ABX) and dextran sulfate sodium (DSS). **b** Weight of DSS-exposed mice (*n* = 12). For statistical comparisons, asterisk (*) indicates ABX versus H_2_O. **c** Length of the colons of mice treated with ABX + *C. albicans*, ABX + *S. boulardii*, or ABX and H_2_O on day 12 (*n* = 12). **d** Secreted lipocalin (pg/mg of feces) at d0, d7, and d12 after DSS (*n* = 6). **e** Representative H&E-stained images of proximal colon cross sections on day 12 after initial DSS exposure. **f** Histological scores on day 12 (*n* = 6). Throughout, data are presented as the mean ± s.e.m. *P < 0.05, **P < 0.01, and ***P < 0.001 by one-way ANOVA with a post hoc Tukey test

To explore the role of the bacterial microbiota in modulating the effects of fungi on colitis, we treated mice with either *C. albicans* or *S. boulardii* daily by gavages and administered broad-spectrum antibiotics (ABX) for 7 days prior to colitis induction with DSS (Fig. 2a). ABX treatment completely protected the mice from colitis and abrogated the impact of fungi on colitis. Only untreated mice exhibited a strong colitis phenotype with significant weight loss (Fig. 2b), reduced colon length (Fig. 2c), high lipocalin levels (Fig. 2d), and histological damage (Fig. 2e, f). Analysis of the microbiota by 16S RNA sequencing showed a strong effect of ABX on the biodiversity and composition of the bacterial microbiota, characterized by a significant reduction of observed species (Additional file 1: Figure S1A and B) and dramatically reduced levels of Firmicutes and Bacteroidetes, along with a dramatic increase in Proteobacteria, which is majorly composed of bacteria from the family *Enterobacteriaceae* (Additional file 1: Figure S1C and D). These results showed that some components of the bacterial microbiota are required for the development of DSS-induced colitis. Consequently, in the absence of these bacteria, colitis does not transpire and pro- or anti-inflammatory fungi challenges do not further bring significant changes in the mice phenotype.

### Vancomycin but not colistin has a protective effect on DSS-induced colitis

**Fig. 3:**
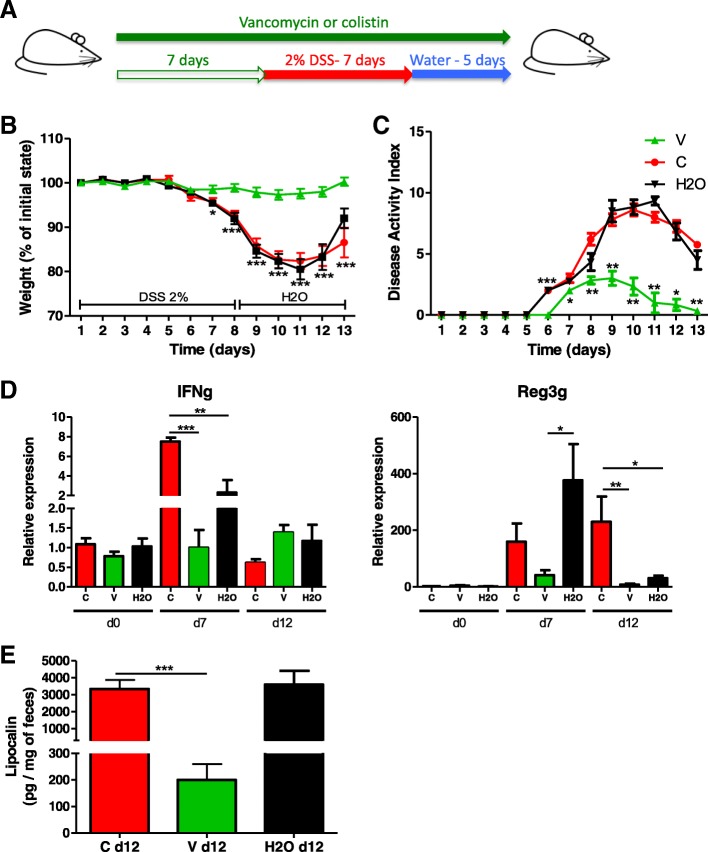
Vancomycin and colistin have different effects on DSS-induced colitis. **a** Experimental design for the administration of vancomycin or colistin antibiotics, and dextran sulfate sodium (DSS). **b** Weight of DSS-exposed mice. For statistical comparisons, an asterisk (*) indicates vancomycin versus colistin/H_2_O. **c** Disease activity index (DAI) of DSS-exposed mice. For statistical comparisons, an asterisk (*) indicates vancomycin versus H_2_O. **d** *IFN-γ* (left) and *Reg3g* (right) transcript expression in the colon after initiation of DSS treatment. **e** Secreted lipocalin (pg/mg of feces) in the feces of mice treated with vancomycin (V), colistin (C), and vehicle (H_2_O). Throughout, data are presented as the mean ± s.e.m. *P < 0.05, **P < 0.01, and ***P < 0.001 by one-way ANOVA with a post hoc Tukey or Dunn’s test; *n* = 12 mice per group from two independent experiments

To evaluate which specific bacterial population in the microbiota affects DSS-induced colitis, we used vancomycin or colistin, which target either gram-positive bacteria or *Enterobacteriaceae*, respectively (Fig. 3a). First, we confirmed the observations made by Nakanishi et al. [29] showing that vancomycin treatment alone recapitulated the strong protective effects of broad-spectrum antibiotics, whereas colistin did not modify the severity of colitis in the treated group compared to the control group. Vancomycin but not colistin reduced DSS-induced pathological changes, such as body weight loss (Fig. 3b), disease activity index (Fig. 3c), colonic transcripts (*IFN-g* and *Reg3g*) and lipocalin levels (Fig. 3d, e), and colon histological scores (Additional file 1: Figure S2A and B), in treated mice compared with control mice. Collectively, these results confirm that certain gram-positive bacteria contribute to the development of DSS-induced colitis.

**Fig. 4:**
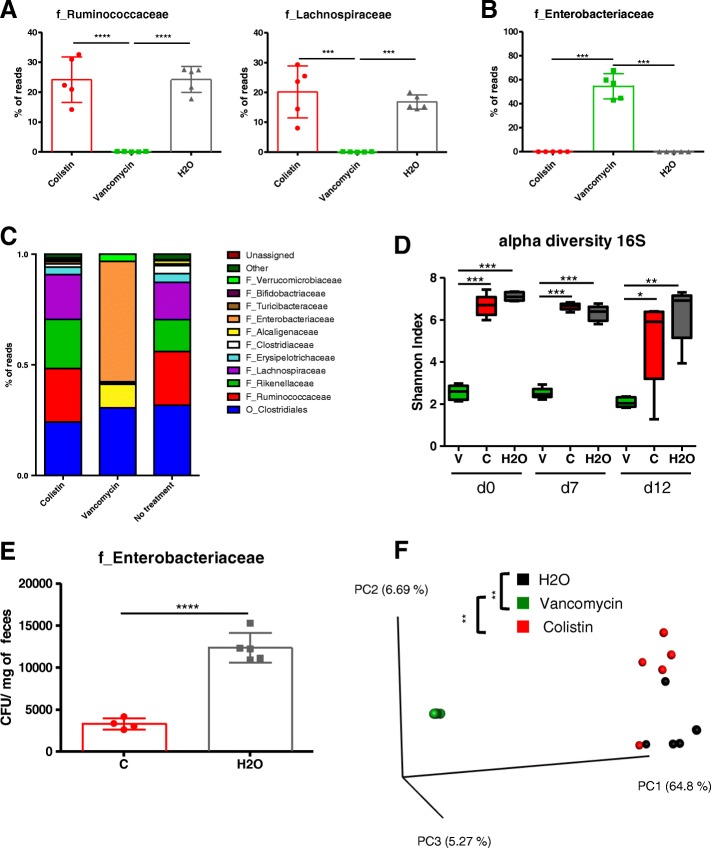
Assessment of microbiota composition after vancomycin or colistin antibiotic treatment. **a** Relative abundance (% reads) of gram-positive *Ruminococcaceae* and *Lachnospiraceae* in the feces of mice treated with colistin, vancomycin, or vehicle at day 0 prior DSS. **b** Relative abundance (% reads) of *Enterobacteriaceae* in the feces of mice treated with colistin compared to untreated mice at day 0. **c** Bacterial-taxon-based analysis at the phylum level in the feces of mice treated with colistin, vancomycin, and vehicle. **d** Shannon index, describing the alpha diversity of the bacterial microbiota (16S) in the fecal microbiota of mice treated with vancomycin (V), colistin (C), and vehicle (H_2_O). **e** *Enterobacteriaceae* levels (CFU/mg of feces) in the feces of mice treated with colistin compared to those of untreated mice. **f** Beta diversity. Principal coordinate analysis of Bray–Curtis distance with each sample colored according to the disease phenotype. PC1, PC2, and PC3 represent the top three principal coordinates that captured most of the diversity. The fraction of diversity captured by the coordinate is given as a percentage. Groups were compared using PERMANOVA. Throughout, data are presented as the mean ± s.e.m. *P < 0.05, **P < 0.01, and ***P < 0.001 by one-way ANOVA with a post hoc Tukey test; *n* = 5 mice per group

We then investigated the effects of vancomycin and colistin on the intestinal microbiota. First, we confirmed using 16S RNA sequencing that vancomycin treatment induced a decrease in gram-positive bacteria such as those from the families *Lachnospiraceae* and *Ruminococcaceae* (Fig. 4a). Similarly to broad-spectrum-treated mice, vancomycin treatment dramatically increases *Enterobacteriaceae* (Fig. 4b, c, Additional file 1: Figure S2C) and significantly reduced bacterial alpha diversity (Shannon index) compared to the colistin and control treatments (Fig. 4d). Colistin showed little effect on the diversity or the global composition of the bacterial microbiota except for the level of *Enterobacteriaceae* (Fig. 4c–e, Additional file 1: Figure S2C). A beta diversity analysis confirmed a relatively weak effect of colistin treatment on the global bacterial microbiota compared to that of the control, while the microbiota after vancomycin treatment formed a cluster significantly separated (*P* value < 0.01) from the microbiota of the two other groups (Fig. 4f). Collectively, these findings showed that vancomycin-sensitive bacteria (consisting essentially of the gram-positive species), but not colistin-sensitive bacteria (consisting essentially of the *Enterobacteriaceae*), are required for the development of DSS-induced colitis.

### Antibiotic treatments alter fungal composition and diversity during colitis

**Fig. 5:**
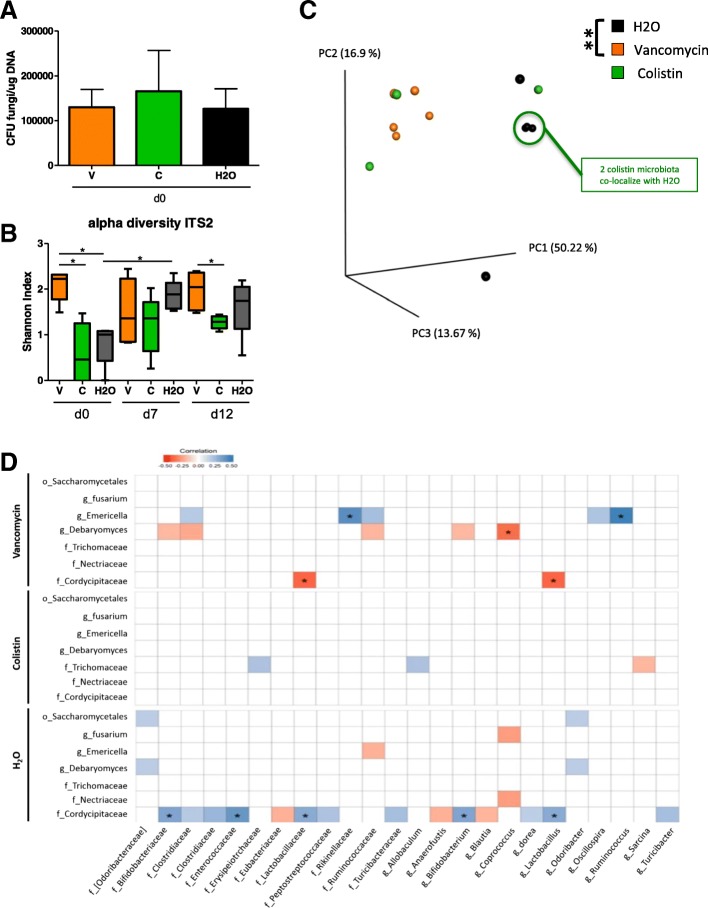
Antibiotic treatments alter fungal microbiota before and during colitis. **a** Fungal levels on day 0 in the fecal microbiota of mice treated with vancomycin, colistin, and vehicle were quantified using 18S rRNA qRT-PCR and were normalized to the bacterial population and quantity of DNA (μg of DNA). Data are presented as the mean ± s.e.m. **b** Shannon index describing the alpha diversity of the fungal microbiota (ITS2) in the fecal microbiota of mice treated with vancomycin (V), colistin (C), and vehicle (H_2_O). **c** Beta diversity. Principal coordinate analysis of Bray–Curtis distance with each sample colored according to the disease phenotype. PC1, PC2, and PC3 represent the top three principal coordinates that, together, captured most of the diversity. The fraction of diversity captured by the coordinate is given as a percentage. Groups were compared using PERMANOVA. **d** Specific bacterial-fungal correlation pattern in mice treated with vancomycin, colistin, and vehicle. Distance correlation plots of the relative abundance of fungal and bacterial families and genera. Statistical significance was determined for all pairwise comparisons; only significant correlations (*P* value < 0.05) are displayed and “*” means that correlations remained statistically significant after correction for false discovery rate. Positive values (blue squares) indicate positive correlations, and negative values (red squares) indicate negative correlations. The shading of the square indicates the magnitude of the association; darker shades are more strongly associated than lighter shades. The sign of the correlation was determined using Spearman’s method. Throughout, data are mean ± s.e.m. *P < 0.05 and **P < 0.01 by one-way ANOVA with a post hoc Tukey test; *n* = 5 mice per group

To evaluate the effects of specific antibacterial treatment on the fungal populations during DSS-induced colitis, we treated mice with vancomycin or colistin and compared their fungal microbiota to that of the untreated control mice. Fungal colonization was homogenous among groups at D0, after antibacterial therapy but before DSS treatment, confirming that in our settings colistin or vancomycin had no direct antifungal effect (Fig. 5a); we also tested that it did not change the global bacterial loads for each condition (Additional file 1: Figure S3A). A detailed examination of the fungal microbiota showed strong differences between groups. The alpha diversity, illustrated by the Shannon index, was significantly increased after vancomycin treatment compared to colistin treatment or no treatment (Fig. 5b). Additionally, beta diversity, illustrating the inter-sample differences, showed a significant modification of the fungal microbiota in vancomycin-treated mice but not colistin-treated mice compared to the control group (Fig. 5c).

Interestingly, these differences, along with the modified bacterial microbiota, had a strong influence on the evolution of the fungal microbiota during DSS treatment (Additional file 1: Figure S3). The equilibrium between fungal phyla also differed between groups: while colistin-treated mice showed a strong decrease in *Ascomycota* at D7 and D12 (Additional file 1: Figure S3b and c), the exact opposite was observed in vancomycin-treated mice, with a nearly complete disappearance of all the *Basidiomycota* (Additional file 1: Figure S3D). Altogether, these data illustrate the presence of strong functional connections between the bacterial and fungal populations in the gut, with an impact on host response in the context of colitis.

### Colistin treatment induces altered correlation between fungal and bacterial relative abundance

In an attempt to elucidate further the interactions between the bacterial and fungal microbiotas, we measured the correlations between the relative abundance of bacterial and fungal taxa at the genus and family levels under antibiotic treatment and compared them to the correlations in untreated mice. We observed an antibiotic-specific pattern with a reduced number of correlations under colistin treatment compared to vancomycin and control; however, none of them showed statistically significant correlation after correction for the false discovery rate (Fig. 5d). In contrast, controls and vancomycin-treated mice showed larger number of correlations, and the strength of those correlations was higher under vancomycin.

Altogether, these data suggest that the presence of colistin-sensitive bacteria strongly influence the bacterial-fungal interactions within the intestine.

### A specific bacterial environment is necessary for the effects of fungi in colitis

**Fig. 6:**
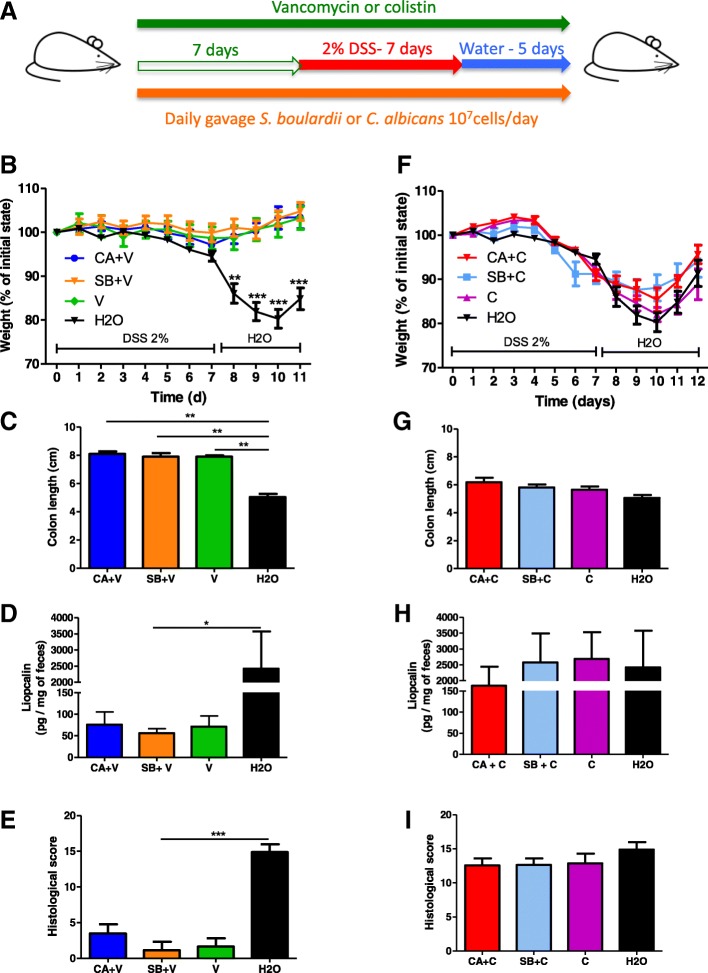
Fungi lose their effects under specific anti-*Enterobacteriaceae* treatment (colistin). **a** Experimental design for the administration of vancomycin or colistin, yeasts (*C. albicans* and *S. boulardii*), and dextran sulfate sodium (DSS). **b** Weight of DSS-exposed mice treated with *C. albicans* + vancomycin, *S. boulardii* + vancomycin, and vancomycin alone (*n* = 12). **c** Length of the colons of mice treated with *C. albicans* + vancomycin (CA + V), *S. boulardii* + vancomycin (SB + V), and vancomycin alone (V) (*n* = 6). **d** Secreted lipocalin (pg/mg of feces) in the feces of mice treated with *C. albicans* + vancomycin, *S. boulardii* + vancomycin, and vancomycin alone (*n* = 6). **e** Histological scores of mice treated with *C. albicans* + vancomycin (CA + V), *S. boulardii* + vancomycin (SB + V), and vancomycin (V) alone on day 12 after DSS (*n* = 6). **f** Weight of DSS-exposed mice treated with *C. albicans* + colistin (CA + C), *S. boulardii* + colistin (SB + C), and colistin alone. **g** Length of the colons of mice treated with *C. albicans* + colistin (CA + C) (*n* = 11), *S. boulardii* + colistin (SB + C) (*n* = 8), and colistin alone (C) (*n* = 7). **h** Secreted lipocalin (pg/mg of feces) in the feces of mice treated with *C. albicans* + colistin (*n* = 12), *S. boulardii* + colistin (*n* = 8), and colistin alone (*n* = 7). **i** Histological scores of mice treated with *C. albicans* + colistin (*n* = 9), *S. boulardii* + colistin (*n* = 9), and colistin alone (*n* = 7) on day 12 after DSS

To assess whether specific bacterial components of the gut microbiota have an impact on the effects of fungi on gut inflammation, mice gavaged daily with *C. albicans* or *S. boulardii* were treated with either vancomycin or colistin for 7 days before colitis was induced with DSS (Fig. 6a). Under vancomycin treatment, all the mice were protected from colitis, showing that gram-positive bacteria are necessary for the onset of colitis, irrespective of the presence of specific fungi (Fig. 6b–e) and Additional file 1: Figure S4A-C). Under colistin treatment, neither *C. albicans* nor *S. boulardii* had any impact on colitis severity, whether at a macroscopic (colon length, weight loss, and histological score) or molecular (lipocalin concentration) level (Fig. 6f–i). Altogether, these results show that the detrimental effects of *C. albicans* and the beneficial effects of *S. boulardii* in colitis depend on the presence of colistin-sensitive bacteria, most likely *Enterobacteriaceae*, within the gut microbiota.

### Supplementation with *E. coli* restores the effects of fungi on colitis in colistin-treated mice

**Fig. 7:**
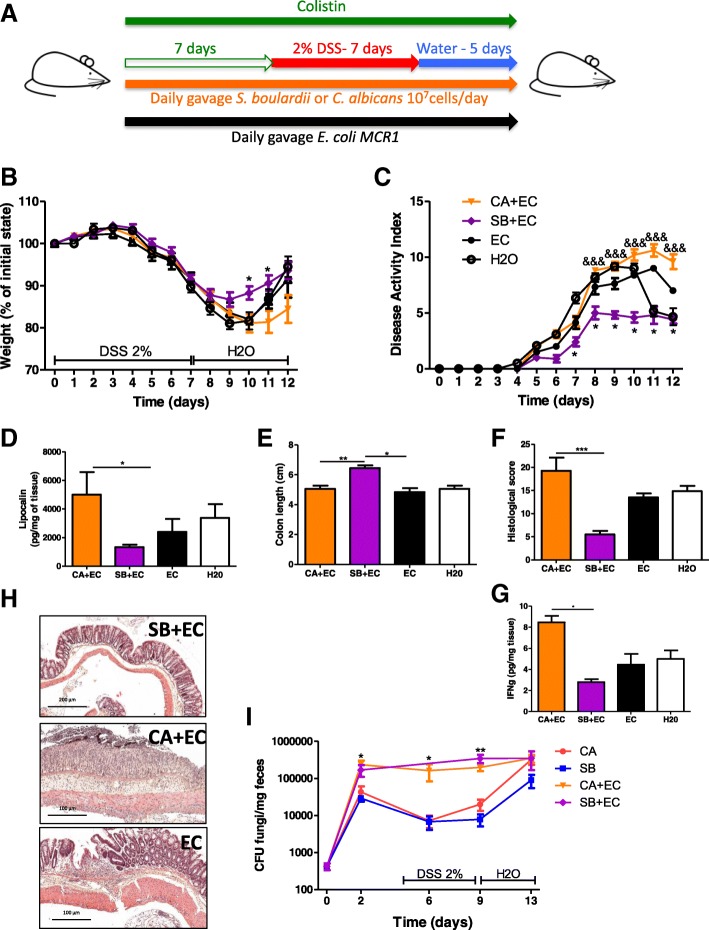
Restoration of the effect of fungi on colitis with *E. coli* supplementation. **a** Experimental design for the administration of *E. coli* MCR1, yeasts (*C. albicans* and *S. boulardii*), colistin, and DSS. **b** Weight of DSS-exposed mice (*n* = 12 per group). For statistical comparisons, an asterisk (*) indicates *C. albicans* + *E. coli* + colistin versus *S. boulardii* + *E. coli* + colistin and *E. coli*. **c** Disease activity index (DAI) of DSS-exposed mice (*n* = 12 per group). For statistical comparisons, asterisk (*) indicates *S. boulardii* + *E. coli* + colistin versus *E. coli* + colistin, and (&) indicates *C. albicans* + *E. coli* + colistin versus *S. boulardii* + *E. coli* + colistin. **d** Secreted lipocalin (pg/mg of feces) in the feces of mice treated with *C. albicans* + *E. coli* + colistin (*n* = 6), *S. boulardii* + *E. coli* + colistin (*n* = 7), and *E. coli* + colistin (*n* = 7). **e** Length of the colons of mice treated with *C. albicans* + *E. coli* + colistin (*n* = 9), *S. boulardii* + *E. coli* + colistin (*n* = 8), and *E. coli* + colistin (*n* = 8). **f** Histological scores of mice treated with *C. albicans* + *E. coli* + colistin (CA + EC) (*n* = 7), *S. boulardii + E. coli* + colistin (SB + EC) (*n* = 7), and *E. coli* + colistin (EC) (*n* = 4) on day 12 after DSS. **g** Amounts (pg/mg tissue) of IFN-γ (left) secreted by colon explants from mice treated with *C. albicans* + *E. coli* + colistin, *S. boulardii* + *E. coli* + colistin, and *E. coli* + colistin. **h** Representative H&E-stained images of proximal colon cross sections on day 12 after initial DSS exposure. **i** *C. albicans* (CA) and *S. boulardii* (SB) counts in the feces of mice treated with colistin or with colistin and *E. coli* (EC) and administered CA and SB (*n* = 5 per group and time point) by gavage. Throughout, data are presented as the mean ± s.e.m. *P < 0.05, **P < 0.01, ***P < 0.001, and ^&&&^*P* < 0.001 by one-way ANOVA with a post hoc Tukey or Dunn’s test

To test whether *Enterobacteriaceae* are necessary for fungi to modulate the severity of colitis, we supplemented colistin-treated mice with a colistin-resistant *E. coli* strain (*E. coli* MCR1) in order to repopulate the gut ecosystem with *Enterobacteriaceae* (Fig. 7a). *E. coli* MCR1 administration alone restored both the beneficial effects of *S. boulardii* and the detrimental effects of *C. albicans* on the severity of colitis as assessed by weight and disease activity index (Fig. 7b, c). On day 7, mice supplemented with *C. albicans* and *E. coli* had higher lipocalin levels than did mice supplemented with *S. boulardii* and *E. coli* (Fig. 7d). Colon length measurements, together with histological scores, also showed a clear improvement when the mice were co-colonized with *S. boulardii* and *E. coli* (Fig. 7e, f, and h). These results were confirmed at a molecular level, showing an increase in pro-inflammatory cytokines such as IFN-γ in the colon of mice treated with *C. albicans* and *E. coli* compared to those treated with *S. boulardii* and *E. coli* (Fig. 7g). To assess the potential effects of *E. coli* on the level of colonization of *C. albicans* and *S. boulardii* in vivo, we quantified each fungus with and without *E. coli* during the course of the experiment. We observed that *E. coli* supplementation in colistin-treated mice was sufficient to promote colonization of *C. albicans* and *S. boulardii*, particularly during the DSS exposition phase (Fig. 7i). Taken together, these results show that *Enterobacteriaceae* are required for the positive and negative effects of fungi in the context of intestinal inflammation. Moreover, the mechanisms involve, at least partly, an ecological effect by which *Enterobacteriaceae* favor intestinal colonization by fungi such as *S. boulardii* and *C. albicans*.

### *Enterobacteriaceae* allow maintenance of fungal loads in the intestine

**Fig. 8:**
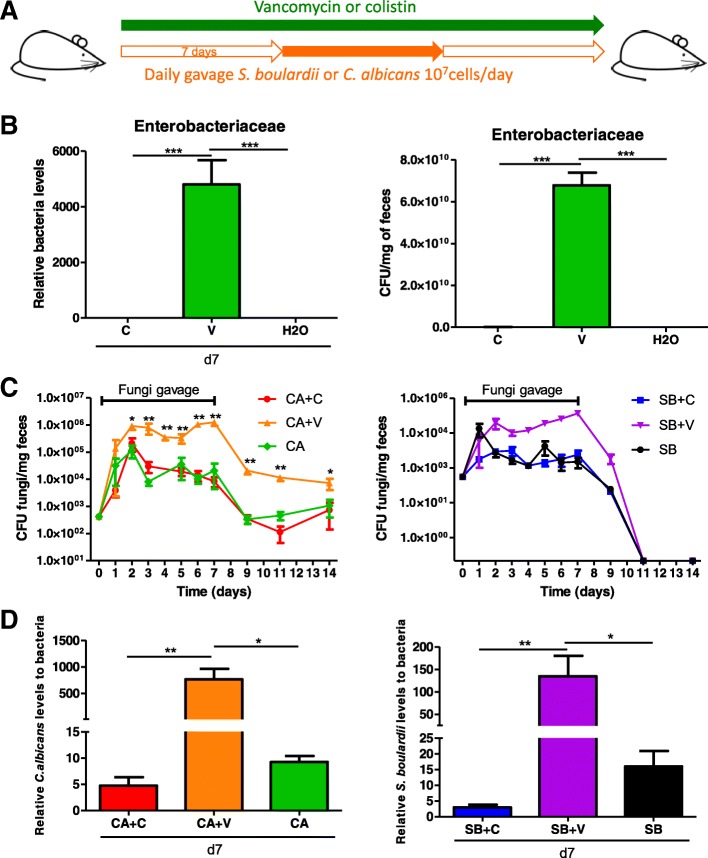
*Enterobacteriaceae* overgrowth increased fungal fitness during gut colonization. **a** Experimental design for the administration of colistin and vancomycin antibiotics and fungal gavages in conventional mice without DSS treatment. **b** Relative (left) and absolute (right) *Enterobacteriaceae* levels in the fecal microbiota of mice treated for 7 days with colistin, vancomycin, or vehicle (PBS) were quantified using 16S RNA qRT-PCR and were normalized to the vehicle group (PBS), day 0. Data are presented as the mean ± s.e.m. (*n* = 5 per group). **c** *C. albicans* (CA) and *S. boulardii* (SB) counts in the feces of mice treated with colistin (C), vancomycin (V), or vehicle (PBS) and administered CA and SB by gavage. For statistical comparisons, an asterisk (*) indicates *C. albicans +* vancomycin versus *C. albicans +* colistin, and (&) indicates *S. boulardii* + vancomycin vs. *C. albicans* + vancomycin (*n* = 5 per group). **d** *C. albicans* levels (left) and *S. boulardii* levels (right) in the fecal microbiota were quantified using specific qRT-PCR primers and were normalized to the bacterial population (*n* = 5 per group). Throughout, data are presented as the mean ± s.e.m. *P < 0.05, **P < 0.01, and ^&&&^*P* < 0.001 by one-way ANOVA with a post hoc Tukey or Dunn’s test

To evaluate the role of bacteria from the family *Enterobacteriaceae* in the ecological fitness of fungi in the gut in a general context, we administered both *C. albicans* and *S. boulardii* to mice treated with vancomycin or colistin but without colitis (Fig. 8a). Under vancomycin, an increased *Enterobacteriaceae* level (both relative and absolute) was observed (Fig. 8b), and both *C. albicans* and *S. boulardii* achieved higher intestinal loads during the daily gavage period in the vancomycin group than in the untreated or colistin-treated group. After the daily gavage was stopped, higher CFU levels of *C. albicans* were still present in vancomycin-treated mice compared to colistin-treated and untreated mice (Fig. 8c). In contrast, *S. boulardii* was rapidly eliminated from the gut after the last gavage in all groups, supporting its reduced adaptation to the gut environment compared to *C. albicans*. The results obtained by culture were confirmed by a specific real-time qPCR approach (Fig. 8d). Taken together, these results suggest that, in a non-colitis context, bacteria from the *Enterobacteriaceae* family cooperate with fungi to favor their gut colonization.

## Discussion

Crosstalk between the gut microbiota and the host is crucial for the creation and maintenance of intestinal homeostasis and is similarly involved in the pathogenesis of diseases such as IBD. Patients with IBD show a strong alteration of their gut microbiota, with a decrease in bacterial diversity and heavy modifications of the equilibrium not only within bacterial and fungal phyla but also possibly in terms of bacterial-fungal inter-kingdom interactions [7]. However, how bacteria and fungi influence each other and whether their interaction can impact microbiota-host homeostasis have been poorly explored to date.

In this study, we investigated the impact of *C. albicans* and *S. boulardii*, two model yeasts, for their effects on gut inflammation, focusing on their interaction with the bacterial microbiota. We showed that the presence of specific bacteria (*Enterobacteriaceae*) is essential for the effect of fungi on gut inflammation, at least partly through their positive influence on fungal colonization.

Although an increase in *C. albicans* and a decrease in *S. cerevisiae* were observed in IBD patients, associated with a bacterial dysbiosis [7, 15, 30, 31], data regarding functional inter-kingdom interactions in inflammatory settings are scarce.

Fungi and bacteria coexist in the environment of the animal gastrointestinal tract; they most likely co-evolved with the hosts over millions of years of animal evolution. The same is true in any other niche, but the gut environment represents a very specific niche with a high concentration of microorganisms that interact extensively with host tissue and immune cells. Our recent sequencing study on a large IBD cohort of 235 patients revealed that positive or negative correlations between fungi and bacteria differ strongly between healthy subjects, Crohn disease (CD) patients, and ulcerative colitis (UC) patients [7]. Specifically, positive and negative correlations between bacteria and fungi in UC patients were higher and statistically stronger than healthy subjects or CD patients displayed. These data suggest that, depending on the localization and the type of inflammation, fungi might not have the same impact. Here, we used antibiotics and yeast challenges to produce disequilibrium between the fungal and bacterial microbiota in order to follow the influence of such an imbalance on the severity of colitis. The initial experiment with broad-spectrum antibiotics highlighted that, in conventional mice, the bacterial microbiota is essential for the development of colitis, since large-scale depletion of the diversity and quantity of bacteria resulted in resistance to DSS colitis. Although early studies reported that broad-spectrum antibiotics worsened DSS-induced colitis [32], our result is in accordance with more recent data by Nakanishi et al. [33]. This controversy illustrates the complexity underlying the initiation and progression of gut inflammation and that antimicrobial drugs might have opposite effects depending on parameters that are not fully understood yet. A simple hypothesis with the current knowledge would be that the basal microbiota composition, which is very likely to be different from one study to another, could influence the effects of antibiotics on the severity of inflammation. Interestingly, in this study, we showed that a pro-inflammatory yeast has negligible inflammatory effects when added in an imbalanced microbiota due to antibiotic treatments but has a potent effect on inflammation if added to the normal microbiota. Thus, *C. albicans*, a well-described opportunistic fungal pathogen and a human gut commensal, requires the presence of specific bacteria that trigger intestinal inflammation in order to increase the intensity. Specific antibiotic treatment with vancomycin or colistin further corroborated these results. Vancomycin was sufficient to recapitulate the effect of broad-spectrum antibiotics, whereas colistin had no visible effect on susceptibility to inflammation. Bacterial microbiota analysis showed that vancomycin profoundly reduced the bacterial alpha diversity and modified the composition of the microbiota, with depletion of gram-positive bacteria and overgrowth of *Enterobacteriaceae*. After vancomycin treatment, mice are completely protected from DSS colitis, and therefore, the protective effects of *S. boulardii* are difficult to assess. On the other hand, colistin only decreased the relative abundance of *Enterobacteriaceae* without vital changes in the microbial diversity. Altogether, these data demonstrated that specific bacteria, most likely gram-positive bacteria of the families *Ruminococcaceae* and *Lachnospiraceae*, are required to trigger inflammation using DSS. On the other hand, colistin-treated mice, with their *Enterobacteriaceae* depleted, exhibited normal susceptibility to colitis but *C. albicans* and *S. boulardii* no longer showed colitis-modulating properties in this setting. Studies of fungal microbiota showed strong disparities between the treated and untreated groups, confirming the indirect effect of bacteria on the fungal population in normal or inflammatory settings. Colistin-treated mice, still sensitive to DSS, showed a strong decrease in *Ascomycota* at D7 and D12 after DSS, a phenotype equally observed during flare-ups in IBD patients [7]. In contrast, the exact opposite was observed in vancomycin treated mice, with the nearly complete disappearance of the *Basidiomycota*. Analysis of specific bacterial-fungal interactions showed an antibiotic-specific pattern with a greatly reduced number of correlations under colistin treatment, which is in accordance with a potential a role of colistin-sensitive *Enterobacteriaceae* in the global interactions between bacteria and fungi within the gut.

Following the reintroduction of *Enterobacteriaceae* (colistin-resistant *E. coli*), *C. albicans* and *S. boulardii* recovered their respective negative and positive effects on the severity of colitis in colistin-treated mice, suggesting that *Enterobacteriaceae* are necessary for the modulatory effect of fungi on gut inflammation. The mechanisms involved might consist, at least in part, of increased fitness of the fungal population in the presence of these bacteria. Our results are, to our knowledge, the first in vivo data documenting an ecological effect of a specific bacterial population on fungal growth and colonization in the gut, with important consequences for gut physiology. *Enterobacteriaceae* have a specific status in the study of gut inflammation and colitis, with publications reporting their role in inducing intestinal inflammation [34]. In our study, a clinical isolate of one colistin-resistant *E. coli* strain was sufficient to strongly modify the fungal population and thus influence the gut ecosystem. These results correlate very well with a recent work by Hoarau et al. studying the microbiota of CD patients and comparing it to that of their non-diseased first-degree relatives [35]; in that study, the authors identified a positive correlation between the presence of *C. tropicalis* and two *Enterobacteriaceae*, *E. coli* and *Serratia marcescens*. In vitro data demonstrated an increase in *C. tropicalis* biofilm formation only with both bacteria and, through microscopic imaging, suggested possible interactions at the cellular level. Other studies have also explored fungal-bacterial interactions in other ecosystems, notably in the lungs of patients with cystic fibrosis, where *Pseudomonas aeruginosa* and *C. albicans* are often identified together [36]. In this particular case, several studies described a detrimental effect of *P. aeruginosa* on *C. albicans* growth through cell-cell interaction and effector secretion [37], pointing out another type of interaction between *Enterobacteriaceae* and fungi.

## Conclusions

In conclusion, our results show a link between the bacterial composition of the microbiota and the gastrointestinal fitness of fungi, as well as their positive or negative effects on inflammation. By manipulating the bacterial composition of the mouse gut microbiota with specific antibiotics, we showed that *Enterobacteriaceae* such as *E. coli* cooperate with yeast to favor their colonization and their active role in intestinal inflammation. These results are a first step toward understanding of the functional connections between fungi and bacteria in the gut. These results also suggest potential therapeutic applications such as potentiating the protective action of a yeast probiotic strain, e.g., *S. boulardii* CNCM I-745, or fighting *C. albicans* infections. For instance, specific targeting of *Enterobacteriaceae* together with anti-fungal drugs could be a promising strategy in patients suffering *C. albicans* overgrowth.

## Additional file

Additional file 1: **Figure S1.** Broad-spectrum antibiotics disrupt microbiota with selection of *Enterobacteriaceae*. **Figure S2.** Vancomycin and colistin have different effects on DSS-induced colitis. **Figure S3.** Effects of antibiotics and DSS treatment on fungal microbiota. **Figure S4.** Gram-positive bacteria are necessary to trigger DSS-induced colitis. (DOCX 1685 kb)

## Abbreviations

ABX: Antibiotics
*C. albicans*: *Candida albicans*
CFU: Colony-forming unit
DAI: Disease activity index
DSS: Dextran sodium sulfate
GIT: Gastrointestinal tract
IBD: Inflammatory bowel disease
IFN: Interferon
IL17a: Interleukin 17a
MCR1: Mobilized colistin resistance 1
PBS: Phosphate-buffered saline
Reg3g: Regenerating islet-derived protein 3 gamma
RPMI: Roswell Park Memorial Institute
*S. boulardii*: *Saccharomyces boulardii*
YEPD: Yeast extract peptone dextrose
