# Supplementary material for: Enterobacteriaceae are essential for the modulation of colitis severity by fungi
Source: Microbiome. 2018 Sep 1;6:152. doi: 10.1186/s40168-018-0538-9 (PMC6119584; doi:10.1186/s40168-018-0538-9)
Supplement: Supplementary file 1 — Figure S1. Broad-spectrum antibiotics disrupt microbiota with selection of Enterobacteriaceae. Figure S2. Vancomycin and colistin have different effects on DSS-induced colitis. Figure S3. Effects of antibiotics and DSS treatment on fungal microbiota. Figure S4. Gram-positive bacteria are necessary to trigger DSS-induced colitis. (DOCX 1685 kb) [file 40168_2018_538_MOESM1_ESM.docx]

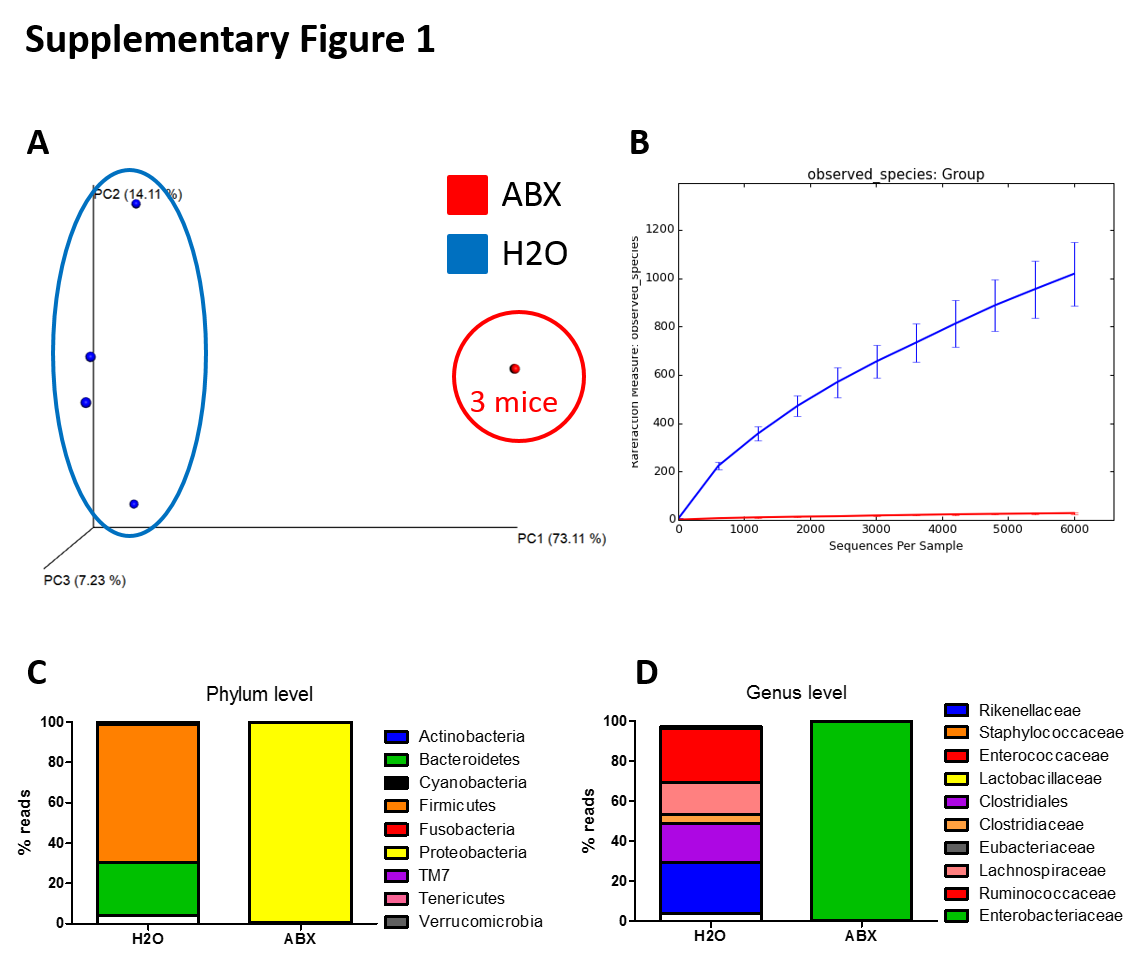


**Figure S1: Broad-spectrum antibiotics disrupt microbiota with selection of *Enterobacteriaceae* and increase of fungal loads**

1. Beta diversity. Principal coordinate analysis of Bray–Curtis distance with each sample colored according to the disease phenotype. PC1, PC2 and PC3 represent the top three principal coordinates that, together, captured most of the diversity. The fraction of diversity captured by the coordinate is given as a percentage. Groups were compared using PERMANOVA.
2. Alpha diversity. Species observed in the fecal samples of mice treated with broad-spectrum antibiotics (red) and in those of untreated mice (blue).
3. Bacterial-taxon-based analysis at the phylum level in the feces of mice treated with broad spectrum antibiotics (ABX) and in those of untreated mice (H_2_O).
4. Bacterial-taxon-based analysis at the genus level in the feces of mice treated with broad spectrum antibiotics (ABX) and in those of untreated mice (H_2_O).


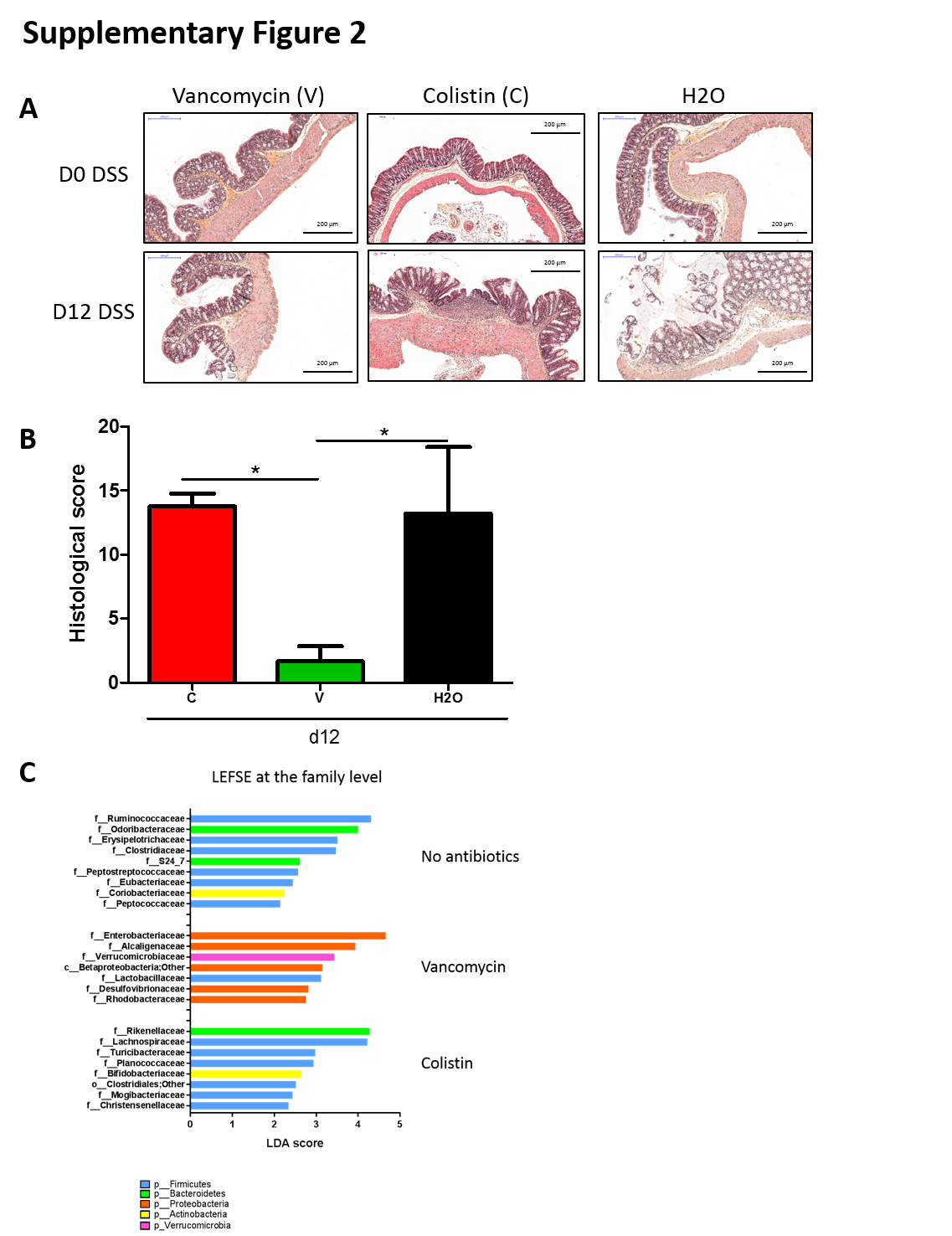


**Figure S2: Vancomycin and colistin have different effects on DSS-induced colitis**

1. Representative H&E-stained images of proximal colon cross-sections on day 12 after initial DSS exposure.
2. Histological scores of colon specimens from mice treated with vancomycin (V), colistin (C) or vehicle (H_2_O) on day 12.
3. Bacterial Family that were differentially represented in the three studied groups with statistical level of significance according to linear discriminant analysis (LDA score >2). The histogram displays all taxa that were increased in each group, as compared with both others, and the corresponding level of significance (LDA score).

Throughout, data are presented as the mean ± s.e.m. **P* < 0.05; ***P* < 0.01; ^***^ P<0.001 by one-way ANOVA with a *post hoc* Tukey or Dunn’s test; *n* = 12 mice per group from two independent experiments.


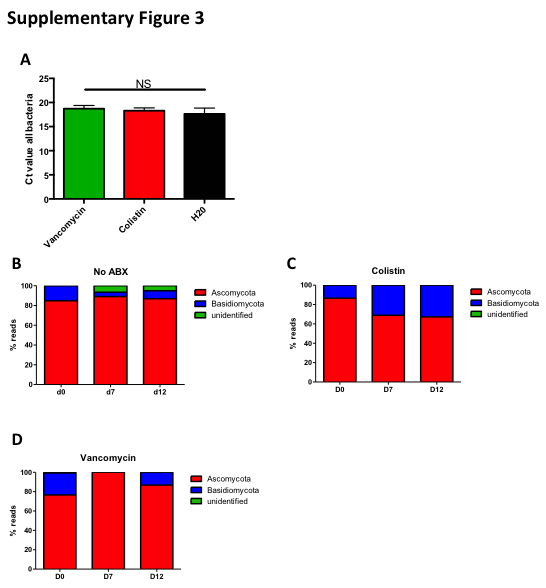


**Figure S3: Effects of antibiotics and DSS treatment on fungal microbiota**

1. Ct values obtained after a qPCR amplifying all bacterial 16S in feces of mice treated with vancomycin (green), colistin (red) and untreated controls H2O (black). Results are presented mean ± SEM.
2. Phylum-level global composition of the fungal microbiota of untreated mice (no ABX).
3. Phylum-level global composition of the fungal microbiota of mice treated with colistin.
4. Phylum-level global composition of the fungal microbiota of mice treated with vancomycin.


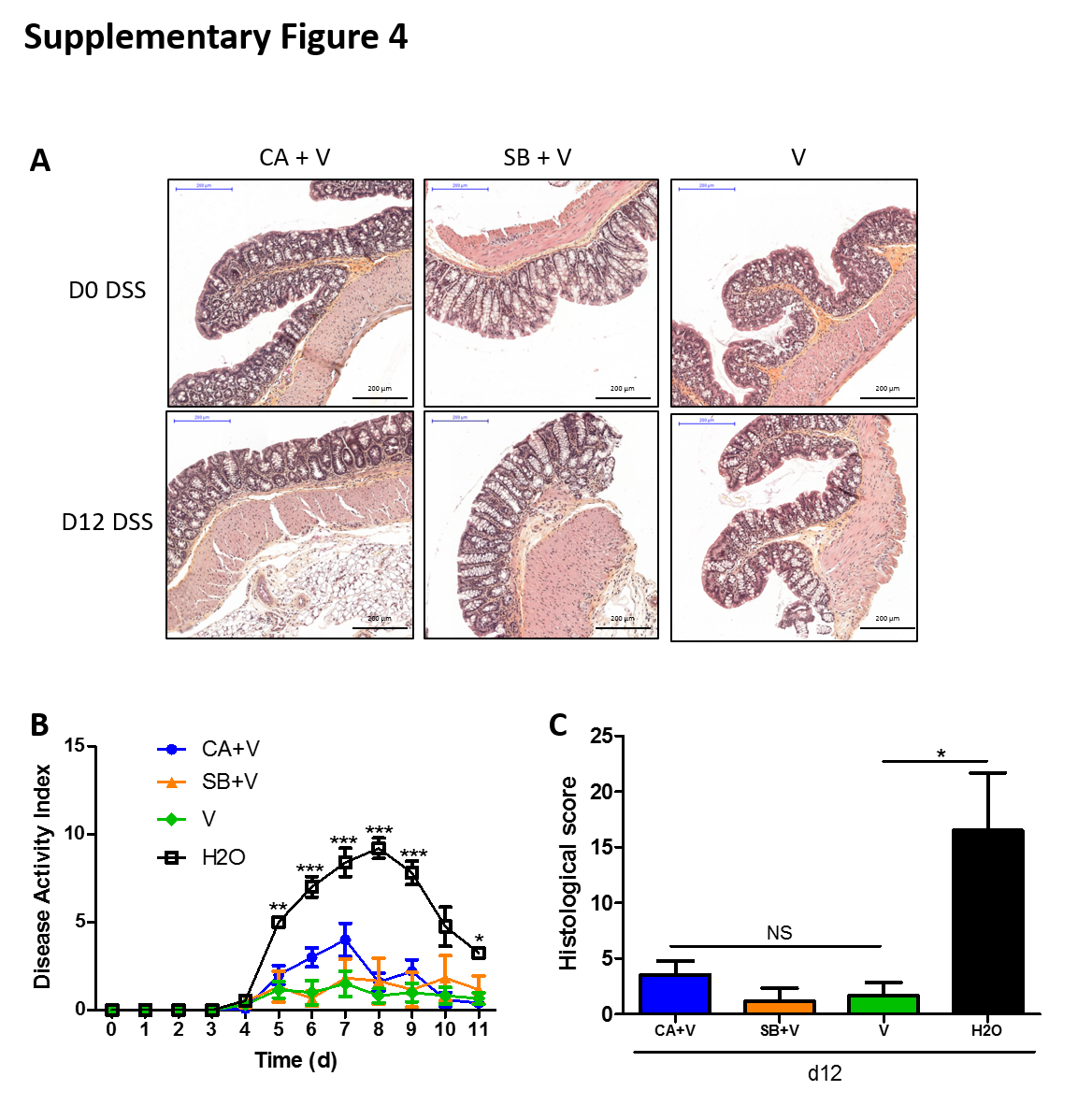


**Figure S4: Gram-positive bacteria are necessary to trigger DSS-induced colitis**

1. Representative H&E-stained images of proximal colon cross-sections on day 12 after initial DSS exposure.
2. Disease activity index (DAI) of DSS-exposed mice. For statistical comparisons, an asterisk (*) indicates untreated mice (H_2_O) versus vancomycin (V)-treated mice.
3. Histological scores at day 12.

Throughout, data are presented as the mean ± s.e.m. **P* < 0.05; ***P* < 0.01; ^***^ P<0.001 by one-way ANOVA with a *post hoc* Tukey or Dunn’s test; *n* = 12 mice per group from two independent experiments.
